# The Ear and Kidney in Mild Scarlet Fever

**Published:** 1902-03

**Authors:** John S. Lankford

**Affiliations:** San Antonio, Texas


For Texas Medical Journal.

### The Ear and Kidney in Mild Scarlet Fever.

BY JOHN S. LANKFORD, M. D., SAN ANTOÑIO, TEXAS.

It is not my intention just now to speak of the pathology, diagnosis, or treatment, of scarlet fever in general, but to emphasize two points in the discussion of the disease as we find it in Southwest Texas, and they both grow out of the climatic modification of the disease.

One is that we are not careful enough to protect the hearing, and the other is that we do not watch the kidneys long enough. I am aware of the fact that we occasionally meet with cases of the most virulent type, terribly destructive to the special senses and to life but the fact remains that the disease usually prevails here in milder form than in any other part of the United States, and that physicians, as well as the people, are too much inclined to underrate its importance, especially with reference to the possible effect upon the ears and the kidneys.

Of thirty-nine cases under my care during the last seven years, only five or six were seriously ill, and all recovered. But in the presence of such trivial indications we should keep in mind the fact that deafness is apt to follow even in mild cases, and that nephritis may occur weeks after all evidence of the disease seems to have vanished.

Two cases that came under my observation will illustrate. F. B., a boy from the country, was under treatment for scarlet fever, of moderate severity, three weeks, when he was sent home. Ten days later I was much mortified to have him brought back, suffering with a mastoid abscess, a ruptured drum, and his hearing permanently impaired to a considerable degree. If this child had been watched for another week or two, and the nose and throat kept

---

*Read before the twenty-fifth annual meeting of the Western Texas Medical Association, at San Antonio, Texas, October 31, 1901.

clean and soothed by proper treatment, particularly in the region of the Eustachian tubes, the danger to life would have been averted and his hearing would have been preserved perfectly.

L. K., a little girl suffering with a sore throat and an eruption, playing round the yard with four or five brothers and sisters all affected alike, was brought in for me to examine in a house where I was attending a case of typhoid fever just convalescing. The disease was diagnosed scarlet fever, and the diagnosis was clearly proven later by the typhoid patient, who had been exposed, developing a typical and nearly fatal attack. The family persisted in neglecting the children, because their illness seemed unimportant, though it was in the fall of the year and the danger emphatically pointed out. Some weeks later the child developed a sub-acute nephritis, which gradually passed into a chronic condition, and the patient has never recovered entirely, and never can. It is a well known fact that there is as much danger in kidney complication in the mild as in the severe form of scarlet fever, and I am convinced that the danger of kidney lesion is far more grave than we have thought in this country, where the disease prevails in such mild form, and where precautions against complications are so much neglected. It would be interesting to follow up these cases which we dismiss so early, or that have no attention at all, and see how many of them suffer with Bright's disease later in life, the chronic disease having its origin in moderate and unknown conditions existing during mild scarlet fever. It is strongly probable that structural conditions of the kidneys occur in many of these mild cases which present no known manifestations, and yet which may lead on to chronic organic disease later.

It is not intended to mention any particular plan of treatment. The point I want to insist on is that even the mildest case of scarlet fever, under the best possible climatic conditions, should be kept under observation for six or eight weeks, and the ear and kidney carefully guarded.

